# Clinical implications of circulating tumor DNA in predicting the outcome of diffuse large B cell lymphoma patients receiving first-line therapy

**DOI:** 10.1186/s12916-022-02562-3

**Published:** 2022-10-25

**Authors:** Miaomiao Li, Lan Mi, Chunyang Wang, Xiaojuan Wang, Jianhua Zhu, Fei Qi, Hui Yu, Yingying Ye, Dedao Wang, Jiaowu Cao, Dingyao Hu, Quanyu Yang, Dandan Zhao, Tonghui Ma, Yuqin Song, Jun Zhu

**Affiliations:** 1 Key Laboratory of Carcinogenesis and Translational Research (Ministry of Education), Department of Lymphoma, Peking University Cancer Hospital & Institute, Beijing, 100142 China; 2 Jichenjunchuang Clinical Laboratory, Hangzhou, Zhejiang China

**Keywords:** Diffuse large B cell lymphoma, Circulating tumor DNA, Minimal residual disease

## Abstract

**Background:**

Circulating tumor DNA (ctDNA) has been proven to be a promising tumor-specific biomarker in solid tumors, but its clinical utility in risk stratification and early prediction of relapse for diffuse large B cell lymphoma (DLBCL) has not been well explored.

**Methods:**

Here, using a lymphoma-specific sequencing panel, we assessed the prognostic and predictive utilities of ctDNA measurements before, during, and after first-line therapy in 73 Chinese DLBCL patients.

**Results:**

The pretreatment ctDNA level serving as an independent prognostic factor for both progression-free survival (PFS, adjusted HR 2.47; *p* = 0.004) and overall survival (OS, adjusted HR 2.49; *p* = 0.011) was confirmed in our cohort. Furthermore, the patients classified as molecular responders who presented a larger decrease in ctDNA levels after the initial two treatment cycles had more favorable PFS (unreached vs. 6.25 months; HR 5.348; *p* = 0.0015) and OS (unreached vs. 25.87; HR 4.0; *p* = 0.028) than non-responders. In addition, interim ctDNA clearance may be an alternative noninvasive method of positron emission tomography and computed tomography (PET-CT) for predicting better PFS (HR 3.65; *p* = 0.0033) and OS (HR 3.536; *p* = 0.016). We also demonstrated that posttreatment ctDNA was a sensitive indicator for detecting minimal residual disease (MRD) in patients with a high risk of recurrence (HR 6.471; *p* = 0.014), who were otherwise claimed to achieve radiographic CR (complete remission).

**Conclusions:**

CtDNA is a promising noninvasive tool for prognosis prediction, response assessment, and early relapse prediction of first-line treatment in DLBCL patients.

**Supplementary Information:**

The online version contains supplementary material available at 10.1186/s12916-022-02562-3.

## Background

Diffuse large B cell lymphoma (DLBCL) is the most prevalent subtype of non-Hodgkin lymphoma (NHL), with high clinical and biological heterogeneity [1, 2]. The use of standard R-CHOP treatment, comprising rituximab, cyclophosphamide, doxorubicin, vincristine, and prednisone has remarkably improved the survival rates of DLBCL patients in the last two decades. However, 30–40% of patients develop relapse or refractory disease [3, 4]. This affirms the need to better stratify DLBCL patients who are unlikely to respond to R-CHOP therapy and develop individualized therapeutic options. At present, identifying patients at high risk of DLBCL and improving their outcomes remain challenges. Although the International Prognostic Index (IPI) is now the most reliable prognostic indicator for DLBCL patients, its effectiveness remains unsatisfactory [5–8]. Previous studies have demonstrated that molecular features detected by tissue biopsy are excellent prognostic biomarkers, but they are invasive and limited to sampling bias [9]. Although radiographic methods are the gold standard for response evaluation and early detection of recurrence, they are neither sensitive nor specific enough and have also been associated with the risk of radiation exposure [10, 11]. Therefore, the development of alternative accessible methods for risk stratification, response assessment, and early relapse identification is imperative for effective management of the disease.

Plasma circulating tumor DNA (ctDNA) has shown great potential as a noninvasive biomarker alternative to tumor tissue biopsy for molecular profiling and has been investigated for prognosis prediction, tracking response to treatment, and predicting disease recurrence in a variety of solid and hematologic tumors [12–16]. Notably, some studies have employed next-generation sequencing to reveal the clinical value of pretreatment ctDNA and ctDNA dynamics during treatment in DLBCL patients [17]. However, the conclusions about the prognostic role of pretreatment ctDNA are controversial [18, 19], and studies on the ctDNA dynamics for therapeutic response prediction are limited in the Caucasian cohorts [19, 20]. Furthermore, the early and accurate detection of minimal residual disease (MRD) through ctDNA profiling to predict relapse has been considered advantageous over traditional imaging scans in multiple solid tumors [21], while ctDNA-based MRD detection in DLBCL remains poorly understood.

In the present study, targeted next-generation sequencing was performed to identify mutations from primary tumor tissue and serial plasma samples collected before, during, and after front-line treatment in a cohort of Chinese DLBCL patients. We explored the clinical value of ctDNA for molecular profiling, disease monitoring, tracking response to treatment, and detecting minimal residual disease.

## Methods

### Patient enrollment

A total of 89 patients who were diagnosed with DLBCL and treated at Peking University Cancer Hospital & Institute between November 2011 and September 2019 were enrolled retrospectively in this study. Each patient’s diagnosis was confirmed by two independent pathologists. Patients were included if they met the following criteria: (1) were radiologically and pathologically diagnosed with DLBCL according to the 2016 World Health Organization classification of lymphoma; (2) were aged ≥ 18 years; (3) had sufficient tumor biopsy tissue and were willing to offer peripheral blood samples before and during treatment cycles, as well as during follow-up; (4) exhibited at least 1 measurable lesion; (5) underwent at least one cycle of first-line chemotherapy at our institute; and (6) underwent at least one treatment response assessment. Conversely, subjects were excluded if they (1) exhibited bone marrow involvement or (2) had severe inflammatory diseases and complications. Prior to being included in the study, all patients signed a written informed consent form.

The clinical and pathological data of each patient, including patient sex, age, stage, B symptoms, lymph node or extranodal invasion, cell-of-origin (COO) classification, lactate dehydrogenase (LDH), IPI, and Eastern Cooperative Oncology Group Performance Status (ECOG PS), were obtained from their medical records. Stages were classified according to the Ann Arbor staging system. All patients were treated with standard chemoimmunotherapy or chemotherapy, while response assessment was performed according to the Lugano response criteria for non-Hodgkin lymphoma. The ethics committee of Peking University Cancer Hospital & Institute approved this study (Approval number: 2017YJZ04).

### Sample collection and processing

Tissue samples were obtained by surgical excision or puncture biopsy before treatment, and stored in a freezer at − 80 °C. Each patient’s serial peripheral blood samples were also obtained prior to and during treatment cycles (at the end of each two chemotherapy cycles) as well as during follow-up. The blood samples were collected into EDTA-coated tubes (BD Biosciences) and centrifuged for 10 min at 1900 g to separate supernatants and white blood cells (WBCs). The supernatants were further centrifuged for 10 min at 16,000 g to isolate plasma, which was subsequently aliquoted into 1.5–2 mL tubes and stored at − 80 °C.

### Extraction of genomic DNA and cell-free DNA

Genomic DNA (gDNA) was extracted from the surgical or biopsy tissues and WBCs using the QIAamp DNA Tissue & Blood Kit (Qiagen), according to the manufacturer's instructions. Cell-free DNA (cfDNA) was extracted from plasma using the MagMAX™ CellFree DNA Isolation Kit (ThermoFisher Scientific). The quality of the extracted cfDNA was assessed using an Agilent 2100 bioanalyzer and the DNA high sensitivity kit (Agilent Technologies). Isolated DNA from each sample was quantified with the Qubit 2.0 Fluorometer using the Qubit dsDNA HS Assay kit (Life Technologies), according to the recommended protocol.

### Targeted DNA sequencing

The Covaris M220 Focused-ultrasonicator™ Instrument (Covaris) was used to shear genomic DNA into 150–200 bp fragments. Next, sequencing libraries were prepared from fragmented gDNA (200 ng) and cfDNA (15–30 ng) using the KAPA HTP Library Preparation Kit (KAPA Biosystems), and duplex unique molecular identifiers (UMIs) were ligated onto the cfDNA fragments. The DNA libraries were captured with the Onco-LymScan panel (Genetron Health) targeting 188 lymphoma-related genes (Additional file 1: Table S1), and the P5/P7 primer was used to amplify the enriched libraries. Quality of the libraries was checked using a 2100 Bioanalyzer while quantification was performed using a Qbit3 and a QPCR NGS library quantification kit (Agilent Technologies). The enriched libraries were sequenced on the NovaSeq6000 NGS platform (Illumina) with a mean coverage depth of at least 100 × for WBCs control samples, 1000 × for tissue gDNAs and 3000 × for cfDNAs after removing duplicates.

### Bioinformatics analyses

Raw sequence reads were demultiplexed to allow for zero barcode mismatches and then subjected to FastQC to determine read quality statistics. Sequence adapters and low-quality regions were removed using Trimmomatic, and then the reads were mapped to the hg19 reference genome using BWA (v 0.7.10). Picard was used to distinguish the PCR duplicates, and GATK was applied for local realignment. Single-nucleotide variants (SNVs) and insertions/deletions (Indels) were called using SAMtools (v0.1.1722), and structural variation calling was performed by Crest (v1.0.25). A mutation in plasma ctDNA was retained when the allele fraction was ≥ 0.1% and the mutated read number was ≥ 3. Additionally, a mutation was called if at least 7 mutated reads and allele frequency ≥ 1% were found in the tissue samples. Next, we evaluated probable strand biases and sequencing errors and then adopted the Integrative Genomics Viewer (Broad Institute, USA) to confirm the accuracy of the called variants. Furthermore, we employed ANNOVAR, Oncotator, and Vep to annotate all mutations for genes and functions, as well as repeating genomic areas. The dbNSFP and Exome Aggregation Consortium (ExAC) databases were used to filter out polymorphic nonsynonymous mutation sites. Germline variants were further filtered out by analyzing the genotyping results of gDNA obtained from paired WBCs of each patient.

### Plasma ctDNA level definition

The ctDNA level was classified as haploid genome equivalents (hGE) per mL of plasma (hGE/mL) and calculated with the following formula: [(the mean VAF for all mutations detected) × cfDNA concentration (pg/mL of plasma)] ÷ 3.3, as previously published by Scherer et al. [22]*.* This value was expressed as a base-10 logarithm (Log hGE/mL).

### Statistical analysis

All statistical analyses were conducted using the IBM SPSS (version 21.0), packages implemented in R (version 3.4.1), and GraphPad Prism (version 8.02) software. The sensitivity of plasma cfDNA genotyping was calculated relative to tumor gDNA (gold standard). The following three survival endpoints were considered: (1) progression-free survival (PFS), defined as the period from the date of first-line treatment start to the date of progression, last follow-up, or death from any reason; (2) relapse-free survival (RFS), defined as the period from the date of first complete response to first progression, last follow-up, or death from any reason; and (3) overall survival (OS), where an event was calculated from the date of diagnosis to the date of death from any reason or last follow-up. A survival analysis was performed by Kaplan–Meier curves, and differences in survival between groups were tested using a log-rank test. A regression analysis of multiple covariates was conducted using the Cox proportional hazards model. Mutation plots were drawn using the OncoPrinter (v1.0.1) and MutationMapper (v1.0.1) tools on the cBioPortal website (http://cbioportal.org). The Mann–Whitney U test was used to test differences in ctDNA levels between the two groups. Data were considered statistically significant when *p* < 0.05.

## Results

### Patient characteristics

**Fig. 1:**
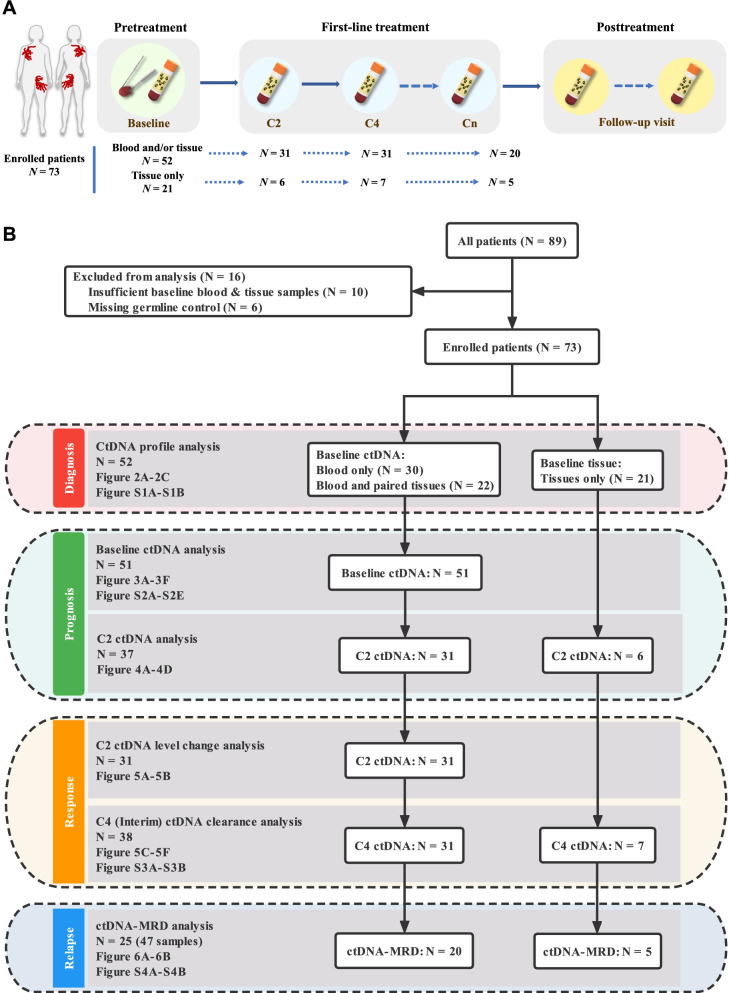
Sample collection and analysis flow chart. **A** Sample collection scheme. Blood and tumor samples collected before treatment were served as the baseline, and C2, C4, and Cn represented on-treatment plasma samples collected at the end of each 2 cycles of therapy. **B** The flowchart shows the samples and patients analyzed in the study. C2, after the second cycle of therapy; C4, after the fourth cycle of therapy; Cn, after the sixth or eighth cycle of therapy; MRD, minimal residual disease

After excluding 6 patients missing normal control samples and 10 patients with insufficient baseline tumor or blood samples, 73 patients were finally included in the analyses in our study. From the 73 patients, we obtained a total of 43 primary tumor specimens (pathologically confirmed) and 162 serial blood samples before, during, and after first-line treatment (Fig. 1A). Profiles of the samples and patients subjected to different analyses are illustrated using a flowchart in Fig. 1B, whereas details on the baseline characteristics of all recruited patients are outlined in Additional file 1: Table S2 and S3. By the last visit in October 2021, the median follow-up duration was 30.3 (range, 3.8–101.2) months. The enrolled cohort comprised 34 (46.6%) and 39 (53.4%) male and female patients, respectively, among whom 58.9% were ≤ 60 years old. Most patients (79.5%) were categorized as the non-GCB subtype based on Hans’ COO classification. A total of 24 patients (32.9%) exhibited B symptoms, while 68.5% of patients were at stage III or IV. More than half (54.8%) of the subjects exhibited elevated levels of serum lactate dehydrogenase, while 42.5% of the patients had an IPI score over 2. In terms of treatment, 64.4% of the patients received the R-CHOP regimen, while the others received the R-CHOP-like plan (for details, see Additional file 1: Tables S2 and S3). After treatment, 44 (60.3%) patients achieved complete remission (CR), 5 (6.8%) achieved partial remission (PR), 1 (1.4%) showed stable disease (SD), and 23 (31.5%) developed progressive disease (PD) rapidly.

### Noninvasive mutational profiling of newly diagnosed DLBCL

**Fig. 2:**
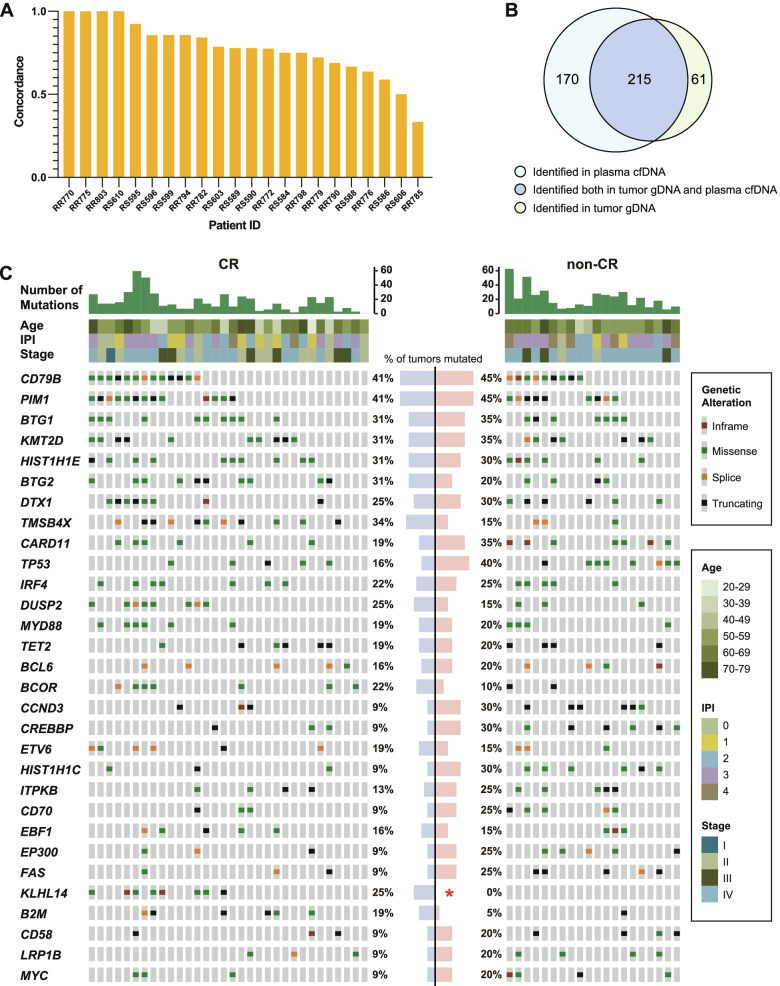
Plasma ctDNA is a reliable source for DLBCL genotyping. **A** The detection concordance between plasma cfDNA and tumor gDNA in detecting mutations for each patient (*N* = 22). **B** Venn diagram shows the number of mutations detected in plasma cfDNA and/or tumor gDNA. **C** Mutation profiles of newly diagnosed DLBCL patients (*N* = 52). A comparison between the CR (*n* = 32) and non-CR (*n* = 20) groups is shown. Each column represents one patient, whereas each row represents one gene. IPI, International Prognostic Index; CR, complete remission; NA, not available

We obtained mean coverage depths of 2392 × (range, 1711 × to 3104 ×) for tumor gDNA and 3953 × (range, 794 × to 7457 ×) for cfDNA samples (Additional file 1: Tables S4 and S5). To establish that plasma cfDNA mirrors tumor tissue in the identification of somatic mutations in DLBCL, we compared the concordance of tumor and ctDNA genotyping among 22 paired baseline tumor/plasma samples. The concordance of each individual is presented in Fig. 2A and the median concordance was 77.8%. Plasma ctDNA achieved an overall sensitivity of 77.9% (215 of 276) in detecting variants verified in tumor specimens (Fig. 2B), indicating that cfDNA is a reliable source for DLBCL genotyping. In addition, ctDNA allowed for the identification of additional 170 somatic mutations that were undetectable in tumor gDNA, which demonstrated that ctDNA could overcome tumor spatial heterogeneity.

Next, we noninvasively characterized the mutational landscape of the 52 patients available with pretreatment plasma samples. Nonsynonymous somatic mutations were detected in 98% (51/52) of the patients with a median of 14 mutations per sample (range, 0–62) and a total of 951 mutations. The most frequently mutated (> 30% in cases) genes in the baseline cfDNA were as follows: *CD79B* (42%), *PIM1* (42%), *BTG1* (33%), *KMT2D* (33%), and *HIST1H1E* (31%) (Additional file 2: Fig. S1A). Among them, *PIM1* and *BTG1* were also identified to be individually hypermutated, which was associated with AID (activation-induced cytidine deaminase) related somatic hypermutation (SHM) as previously described [23–25]. Mutation landscape of tumor gDNA was performed on baseline tumor biopsies from 43 patients. The most frequently mutated genes in the tumor tissue cohort were *DTX1* (37%), *CD79B* (35%), *BTG1* (30%), *BTG2* (30%), and *TMSB4X* (30%) (Additional file 2: Fig. S1B).

We then evaluated whether individual mutations were associated with the response to treatment. In summary, we stratified patients into two groups, namely CR (*n* = 32) and non-CR (*n* = 20), and then compared the baseline mutation profiles between the two groups (Fig. 2C). The *KLHL14* gene presented significantly different mutation frequencies between them (CR group and non-CR group, 25% vs. 0%, *p* = 0.015, Fisher's exact test), indicating that *KLHL14* may serve as a predictive marker for CR achievement upon front line therapy. Correspondingly, mutation frequencies of the *KLHL14* gene among the 43 patients with primary tumor tissue samples revealed a similar trend, although the difference was not significant (*p* = 0.064), possibly due to a limited sample size.

### Pretreatment ctDNA level is an independent prognostic biomarker

**Fig. 3:**
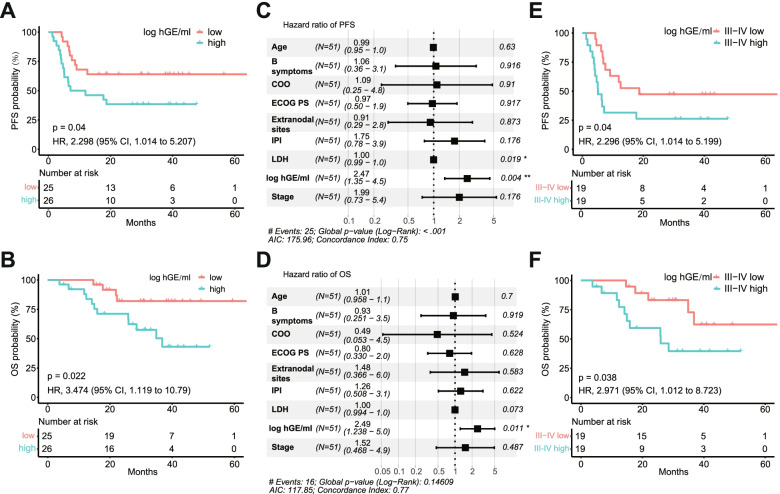
Pretreatment ctDNA level is an independent prognostic biomarker for DLBCL patients (*N* = 51). **A**, **B** Kaplan–Meier estimates of PFS and OS based on pretreatment ctDNA levels. **C**, **D** Multivariable cox proportional hazard models of PFS and OS based on pretreatment ctDNA levels and other survival predictors. **E**, **F** Kaplan–Meier estimates showing the impact of pretreatment ctDNA levels on PFS and OS in patients with III–IV stage (*N* = 38). PFS, progression-free survival; OS, overall survival; hGE, haploid genome equivalents; HR, hazard ratio; COO, cell of origin; LDH, lactate dehydrogenase

Previous studies have shown that baseline ctDNA levels are effective in predicting the survival of DLBCL patients [18, 19, 22]. However, until now, its prognostic value in the Chinese cohort remains unclear. A total of 51 patients were included in the survival analysis by excluding patient RR584, who was lost to follow-up after treatment. The cohort obtained a median ctDNA level of 2.44 log hGE/ml (range, not detected to 4.93). The analysis found that the pretreatment ctDNA levels were significantly associated with several known prognostic factors, including baseline LDH (*p* = 0.0044), IPI score (*p* = 0.0053), B symptoms (*p* = 0.0007), and Ann Arbor stage (*p* = 0.0107), indicating its role as an alternate for disease burden (Additional file 2: Fig. S2A-S2D). Further exploration showed that patients with high ctDNA levels (≥ median, *n* = 26) presented unfavorable progression-free survival (9.5 months vs. unreached; HR 2.298; 95% CI 1.014–5.207; *p* = 0.04; Fig. 3A) and overall survival (36.8 months vs. unreached; HR 3.474; 95% CI 1.119–10.79; *p* = 0.022; Fig. 3B) compared with those with low ctDNA levels (< median, *n* = 25). In Cox univariate analysis, IPI score, stage, extranodal sites, and ctDNA level showed prognostic value for survival (Additional file 1: Table S6). However, in the Cox multivariate analysis, only the pretreatment ctDNA level remained a significant independent prognostic factor for both PFS (HR 2.47; 95% CI 1.35–4.5; *p* = 0.004; Fig. 3C) and OS (HR 2.49; 95% CI 1.238–5.0; *p* = 0.011; Fig. 3D). In addition, precise prognosis stratification is strictly necessary in advanced-stage patients, as the outcomes of these patients were highly variable. In this study, stage III-IV patients (*N* = 38) with higher ctDNA levels (above median, *n* = 19) have poor PFS (5.1 months vs. 18.7 months; HR 2.296, 95% CI 1.014–5.199; *p* = 0.04; Fig. 3E) and OS (26.0 months vs. unreached; HR 2.971; 95% CI 1.012–8.723; *p* = 0.038; Fig. 3F) than their counterparts with lower ctDNA levels (below median, *n* = 17). Moreover, when correlated with therapeutic response, patients who developed PD after the first-line standard treatment showed higher baseline ctDNA levels (*p* = 0.0002; Additional file 2: Fig. S2E) than those who did not. Taken together, our results demonstrated that the pretreatment ctDNA level is a robust independent biomarker for risk stratification and outcome prediction in DLBCL patients.

### CtDNA levels after the second cycle of treatment also effectively predict survival

**Fig. 4:**
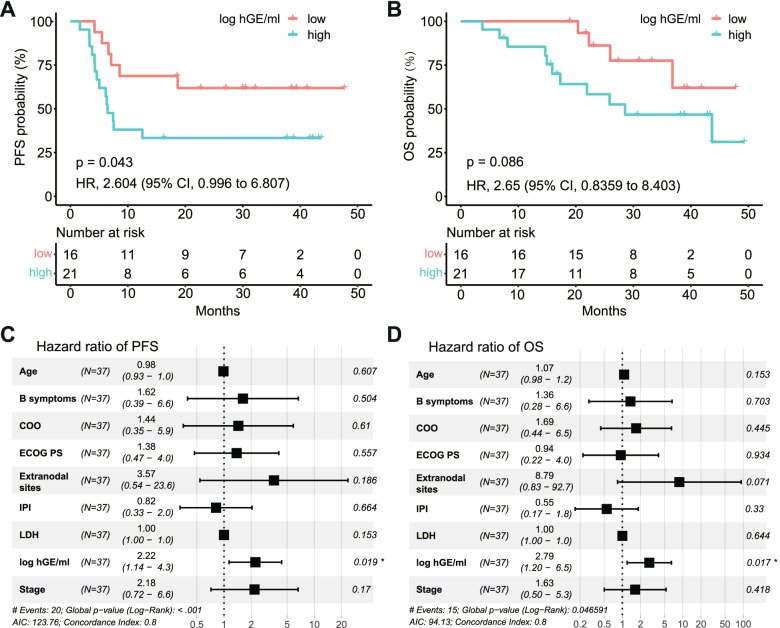
High ctDNA level at C2 is correlated with poor survival (*N* = 37). **A**, **B** Kaplan–Meier curves of PFS (**A**) and OS (**B**) based on C2 ctDNA levels. **C**, **D** Multivariable cox proportional hazard models for PFS and OS based on C2 ctDNA levels and other survival predictors

Although the baseline ctDNA level is a prognostic factor for patient outcome, a fairly high proportion of the enrolled patients (28.7%, 21/73, Fig. 1) in our study did not have pretreatment blood samples. Further explorations are needed to ascertain whether or not ctDNA obtained during treatment is a prognostic biomarker. Therefore, we explored the prognostic value of ctDNA levels after 2 treatment cycles (C2) in 37 patients. Specifically, based on the median ctDNA level (0.9 log hGE/mL; range, not detected to 4.1), we divided the 37 patients into two subgroups, and found a significant difference between them with regard to PFS (HR 2.604; 95% CI 0.996–6.807; *p* = 0.043; Fig. 4A) and a similar trend to OS (HR 2.65; 95% CI 0.8359–8.403; *p* = 0.086; Fig. 4B). Notably, patients with higher ctDNA levels at C2 had a shorter median PFS (6.5 months vs. unreached) and significantly inferior 24-month PFS rates (35% vs. 65%). The multivariate analysis showed that the ctDNA levels at C2 could serve as an independent prognostic factor for both PFS (HR 2.22; 95% CI 1.14–4.3; *p* = 0.019; Fig. 4C) and OS (HR 2.79; 95% CI 1.20–6.5; *p* = 0.017; Fig. 4D).

### CtDNA dynamics during therapy is an alternative noninvasive method to PET-CT for response assessment

**Fig. 5:**
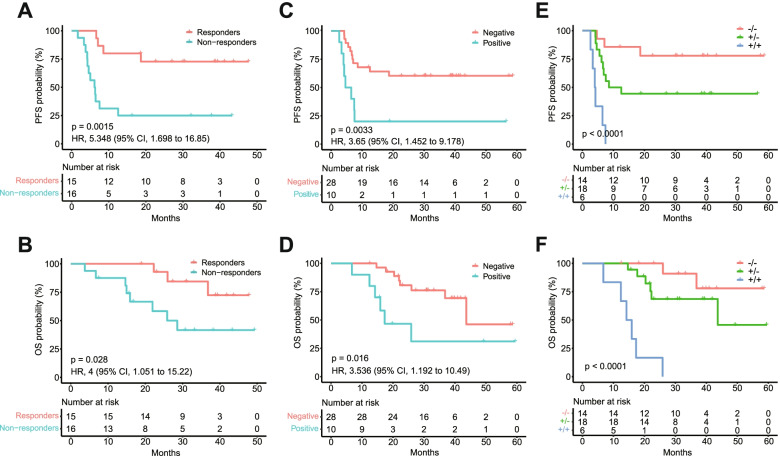
Response prediction value of ctDNA dynamics during treatment. **A**, **B** Kaplan–Meier estimates of PFS (**A**) and OS (**B**) according to the percent changes of C2 ctDNA level relative to baseline (*N* = 31). **C**, **D** Kaplan–Meier estimates of PFS (**C**) and OS (**D**) according to interim ctDNA clearance status at the end of C4 (*N* = 38). **E**, **F** Kaplan–Meier estimates show the PFS (**E**) and OS (**F**) of patients based on the combination of interim PET-CT and ctDNA clearance status (*N* = 38). Patients are divided into three groups: negative interim PET-CT and ctDNA clearance (− / −), positive interim PET-CT and positive ctDNA (+ / +), and either positive interim PET-CT or positive ctDNA (+ / −)

Although radiographic methods are currently the gold standard for response evaluation, they are not sufficiently sensitive and pose a risk of radiation exposure. To reveal the role of ctDNA in response assessment, we profiled the plasma samples collected after the initial and middle cycles during the first-line treatment. With the completion of two treatment cycles, most patients (29/31) had a rapid decrease in ctDNA levels (log hGE/mL), except for two patients whose ctDNA levels were increased in our study. We determined the magnitude of decrease from baseline for each patient (*N* = 31) by the ctDNA level change percent relative to the baseline ctDNA level according to the following formula: (C2—baseline)/baseline. The ctDNA level change percent values varied from − 100.00 to 117.10%, with a median of − 62.27%. Based on the median value, patients were stratified into molecular responder (< median value, *n* = 15) and non-responder (≥ median value, *n* = 16) subgroups. Consequently, the survival analysis indicated that the molecular responders had longer PFS (unreached vs. 6.25 months; HR 5.348; 95% CI 1.698–16.85; *p* = 0.0015) and OS (unreached vs. 25.87 months; HR 4.0; 95% CI 1.051–15.22; *p* = 0.028) than non-responders (Fig. 5A, B). Together, for the first time, these results indicated that early ctDNA dynamics are effective in predicting response during therapy in a Chinese DLBCL cohort.

To explore the role of ctDNA for interim response assessment, we analyzed the interim plasma samples (collected after four cycles of therapy, C4) obtained from 38 patients with paired baseline tumor or plasma samples in which at least one mutation was detected. Tracking these basal mutations in these interim plasma samples revealed ctDNA positive results in 10 patients but negative results in the remaining 28. Patients with detectable ctDNA (positive) in interim plasma had a worse PFS (HR 3.65; 95% CI 1.452–9.178; *p* = 0.0033) and OS (HR 3.536; 95% CI 1.192–10.49; *p* = 0.016) than their counterparts with negative results (Fig. 5C, D). The multivariate analysis revealed that interim ctDNA clearance was an independent prognostic marker in the context of interim PET-CT, and ctDNA presented a comparable prognostic performance to interim PET-CT for PFS (HR 4.7 vs. 4.7, Additional file 2: Fig. S3). When combining interim PET-CT and ctDNA, prediction of survival was remarkably improved. Patients with both negative interim ctDNA and interim PET demonstrated excellent outcomes while patients with both positive interim PET and ctDNA were correlated with extremely poor prognosis (Fig. 5E, F).

### CtDNA monitoring predicts early relapse for DLBCL

**Fig. 6:**
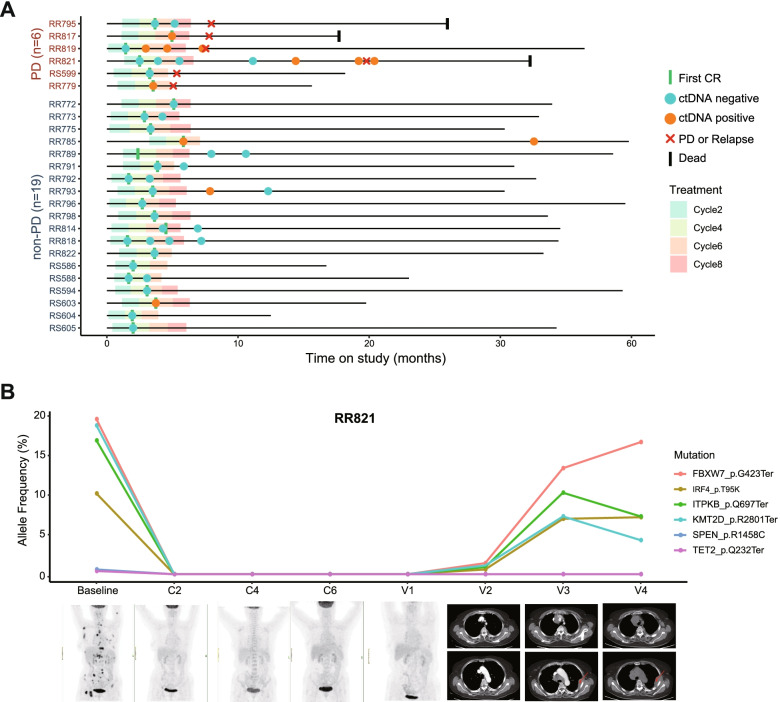
CtDNA is a potential biomarker for MRD detection and relapse prediction. **A** CtDNA monitoring results at different time points in 25 patients who achieved radiographic CR. **B** Gene alterations analyzed using plasma cfDNA at baseline, C2, C4, C6, and follow-up visits for patient RR821 are shown in detail. Radiographic images of the corresponding time points are shown below. RFS, relapse-free survival; PD, progression disease; V1, the first follow-up visit; V2, the second follow-up visit; V3, the third follow-up visit; V4, the fourth follow-up visit

To evaluate the clinical value of ctDNA in detecting MRD in DLBCL, we analyzed the longitudinal plasma samples from 25 patients who reached radiographic CR during or after the first-line treatment. Among them, 19 (76.0%, 19/25) patients survived free of disease during the follow-up, and 6 patients experienced PD or relapse. Their ctDNA status at different time points are presented using a swimmer plot in Fig. 6A. CtDNA-MRD positive was defined as any basal mutation that could be detected in any of the plasma samples at CR status; otherwise, it was termed ctDNA-MRD negative. Only 2 of the 18 ctDNA-MRD negative (11.1%, 2/18) patients developed recurrence or progression disease. However, 4 of the 7 (57.1%, 4/7) ctDNA-MRD positive patients exhibited progression or relapse. The survival curves demonstrated that relapse-free survival (RFS) was significantly poorer in ctDNA-MRD positive patients than in ctDNA-MRD negative patients (median RFS 17.3 months vs. unreached; HR 6.471; 95% CI 1.177–35.58; *p* = 0.014; Additional file 2: Fig. S4A). A similar, albeit insignificant, trend was also observed with regard to OS (HR 6.515; 95% CI 0.588–72.22; *p* = 0.079; Additional file 2: Fig. S4B).

Among the 6 patients who developed PD during follow-up, 2 had available serial plasma samples during surveillance and were subjected to further analysis. Patient RR821, who had baseline plasma mutations in 6 genes (*FBXW7*, *IRF4*, *ITPKB*, *KMT2D*, *SPEN*, and *TET2*), achieved radiographic CR after 2 cycles of R-CHOP regiment treatment and maintained radiographic CR until the end of treatment. Correspondingly, no ctDNA mutation was detected at C2, C4, C6, or the first follow-up visit (V1) timepoints (Fig. 6B). However, ctDNA-MRD turned positive at V2 while CT scans did not show any signs of clinical recurrence until V3/V4, with a gap of 4.8 months. These results demonstrated the high sensitivity of ctDNA in MRD detection for the early and noninvasive prediction of recurrence.

## Discussion

The clinical roles of ctDNA for the management of DLBCL patients undergoing first-line therapy were systematically evaluated by targeted next-generation sequencing in this report. The analysis of tumor biopsies and a series of peripheral blood samples from 73 patients revealed that ctDNA shows robust performance in different clinical courses of the disease. First, noninvasive mutational profiling by plasma ctDNA was feasible. Second, the pretreatment ctDNA level was an independent prognostic biomarker, suggesting that ctDNA could improve risk stratification before treatment. Notably, the ctDNA level after two treatment cycles was also a prognostic factor. Third, it was evident that ctDNA dynamics were significantly associated with clinical outcomes. The percent change in ctDNA levels after C2 relative to baseline was a significant predictor of late long-term outcomes; moreover, interim ctDNA could be a complementary tool for interim PET scans. Last, MRD detection by ctDNA surveillance could effectively predict recurrence ahead of imaging scans.

In this study, at least one mutation was detected in pretreatment plasma in 98% of the DLBCL cases, demonstrating the potentially universal applicability of ctDNA mutation detection. This finding was consistent with previous studies as the mutation frequency varies from 63 to 99% [18, 19, 26–28]. Moreover, the mutation profile of DLBCL mutations obtained in this study was mostly consistent with previously reported signatures [18, 29–31]. Although *KLHL14* is recurrently mutated in mature B cell malignancies, its molecular mechanisms and functions in DLBCL are poorly understood. Until recently, Choi et al. reported that the *KLHL14* gene is a novel tumor suppressor in regulating NF-κB signaling, and inactivation of *KLHL14* leads partial and relative resistance to the Bruton tyrosine kinase (BTK) inhibitor ibrutinib [32]. Of note, in our cohort, the mutation frequency of *KLHL14* was higher in patients reaching CR than those with non-CR treated with first-line treatment, which indicates the potential favorable impact of *KLHL14* mutations in DLBCL treatment. Based on the high mutation frequency, potential clinical value and the lack of knowledge of *KLHL14* in DLBCL, More research on *KLHL14* need to be performed in the future. Taken together, ctDNA profiling is informative and reliable to provide valuable molecular information about DLBCL, especially in the absence of tumor biopsy.

Until now, identifying high-risk DLBCL patients to improve outcomes remains a challenge even though biomarkers such as IPI or COO classification can indicate the prognosis to a certain extent [8]. Despite various studies demonstrating the pretreatment ctDNA’s prognostic utility, its role as an independent prognostic factor remains controversial. For example, while Kurtz et al. [19] indicated that the pretreatment ctDNA level was an independent prognostic marker for event-free survival (EFS), other research evidence reported contrasting results [18], in which the pretreatment ctDNA levels did not maintain a prognostic value for both PFS and OS in multivariate analyses. The results from the present study showed that high pretreatment ctDNA level was a high-risk factor, and it remained an independent prognostic biomarker for both PFS and OS after multivariable analyses in the context of IPI, COO, stage, and other factors. In addition, pretreatment ctDNA can also provide refined prognostic stratification for advanced patients with poor outcomes. Furthermore, the ctDNA levels after the second cycle of therapy can be used as a prognostic biomarker when baseline sampling is not available. Identification of this highest-risk group could provide an opportunity for early intervention with alternative treatment regimens, including new targeted therapies and bone marrow transplantation. Further research explorations for integrating ctDNA with the known risk-stratification methods are expected to validate these findings.

Early identification of non-responders to initial treatment is of great value in clinical practice. Kurtz et al. [19] showed that major molecular response (defined based on the change of a 2.5-log decrease in ctDNA level after two cycles of therapy) was an early outcome prediction biomarker for DLBCL patients undergoing front-line or salvage therapy. Consistently, we found that molecular responders defined by a larger percentage decrease in ctDNA levels after the initial 2 cycles of treatment had better outcomes than nonresponders. To our knowledge, this is the first study describing the predictive role of ctDNA early changes during treatment in a Chinese cohort. The dynamics of ctDNA after 2 cycles showed potential to be an early outcome predictor for later benefits and thus is expected to help clinicians in identifying patients who are unlikely to benefit from the initial cycle of therapy such that the treatment strategies can be adjusted earlier.

Although interim PET-CT is considered a predictor of outcomes [33], alternative methods for the interim assessment of DLBCL patients are needed owing to the low specificity and sensitivity of PET-CT [34, 35]. The correlation of ctDNA detection with survival in DLBCL patients has been reported sporadically, but the conclusions are conflicting. Roschewski et al. [20]retrospectively analyzed the prognostic implications of interim ctDNA by the immunoglobulin high-throughput sequencing (IgHTS) method among 108 DLBCL patients and demonstrated the complementary role of ctDNA for interim PET scans. However, Meriranta L et al. indicated that ctDNA at mid-staging could not predict the failure-free survival of DLBCL patients [36]. In the present study, we employed a targeted gene sequencing panel and found that detectable interim ctDNA is a biomarker of poor prognosis. In addition, the combination of interim ctDNA and PET-CT results in an improved method for the interim assessment.

The potential clinical applications of ctDNA for the detection of radiographically occult MRD have been explored by an increasing number of studies. However, previous related studies have focused largely on solid tumors. To the best of our knowledge, only two studies have explored the possibility of ctDNA-MRD detection for DLBCL. Nevertheless, both studies had a limited number of patients and focused on the analysis of recurrent patients. Whether ctDNA can be used for the detection of MRD in DLBCL patients is still unclear [22, 37]. In the present study, 57.1% ctDNA-MRD positive patients exhibited disease progression or relapse; comparatively, only 11.1% ctDNA-MRD negative patients did. These ctDNA-MRD negative patients exhibited molecular remission to treatment and had better median RFS (unreached vs. 17.3 months) than the ctDNA-MRD positive patients. These findings indicated that ctDNA could not only track MRD during or after the first-line treatment but is also a promising noninvasive biomarker for predicting the risk of recurrence in DLBCL. Further research studies on MRD using large sample sizes and more serial follow-up samplings are urgently needed.

## Conclusions

In conclusion, both pretreatment and dynamic measurements of ctDNA are feasible and can stratify the risk and predict outcomes of DLBCL patients receiving first-line treatment. Future prospective studies are expected to verify the application of these approaches to patients with DLBCL.

## Supplementary Information

**Additional file 1: Table S1.** Gene list of Onco-LymScan panel. **Table S2.** Overview of patient characteristics. **Table S3.** Demographic of patient characteristics. **Table S4.** Sequencing quality data of tumor gDNA samples. **Table S5.** Sequencing quality data of cfDNA samples. **Table S6.** Univariate Cox proportional hazard regression survival analysis including pretreatment ctDNA levels and other clinical indices.

**Additional file 2: Fig. S1.** Mutation profiles of newly diagnosed DLBCL patients based on (A) plasma ctDNA (*N* = 52) and (B) tumor gDNA (*N* = 43) in this cohort. **Fig. S2.** Correlation between pretreatment ctDNA levels and 1) known prognostic factors, including baseline LDH (A), IPI score (B), B symptoms (C), Ann Arbor stage (D), 2) the response to the first-line treatment (E). Undetectable ctDNA was assigned the value of 0 Log hGE/mL. **Fig. S3.** Multivariable cox proportional hazard models for PFS and OS based on interim ctDNA and PET-CT. **Fig. S4.** Kaplan-Meier estimates of RFS (A) and OS (B) according to the ctDNA-MRD status.

## Data Availability

The datasets used in this study are available from the corresponding author on reasonable request.

## Abbreviations

cfDNA: Cell-free DNA
COO: Cell of origin
CR: Complete remission
ctDNA: Circulating tumor DNA
DLBCL: Diffuse large B cell lymphoma
ECOG PS: Eastern Cooperative Oncology Group Performance Status
EDTA: Ethylenediaminetetraacetic acid
ExAC: Exome Aggregation Consortium
gDNA: Genomic DNA
hGE: Haploid genome equivalents
HR: Hazard ratio
Indel: Insertion or deletion event
IPI: International prognostic index
LDH: Lactate dehydrogenase
MRD: Minimal residual disease
OS: Overall survival
PD: Progressive disease
PET-CT: Positron emission tomography and computed tomography
PFS: Progression-free survival
PR: Partial remission
RFS: Relapse-free survival
SD: Stable disease
SHM: Somatic hypermutation
SNV: Single nucleotide variant
WBCs: White blood cells
