# Supplementary material for: Clinical implications of circulating tumor DNA in predicting the outcome of diffuse large B cell lymphoma patients receiving first-line therapy
Source: BMC Med. 2022 Oct 25;20:369. doi: 10.1186/s12916-022-02562-3 (PMC9594942; doi:10.1186/s12916-022-02562-3)
Supplement: Supplementary file 2 — Additional file 2: Fig. S1. Mutation profiles of newly diagnosed DLBCL patients based on (A) plasma ctDNA (N = 52) and (B) tumor gDNA (N = 43) in this cohort. Fig. S2. Correlation between pretreatment ctDNA levels and 1) known prognostic factors, including baseline LDH (A), IPI score (B), B symptoms (C), Ann Arbor stage (D), 2) the response to the first-line treatment (E). Undetectable ctDNA was assigned the value of 0 Log hGE/mL. Fig. S3. Multivariable cox proportional hazard models for PFS and OS based on interim ctDNA and PET-CT. Fig. S4. Kaplan-Meier estimates of RFS (A) and OS (B) according to the ctDNA-MRD status. [file 12916_2022_2562_MOESM2_ESM.docx]

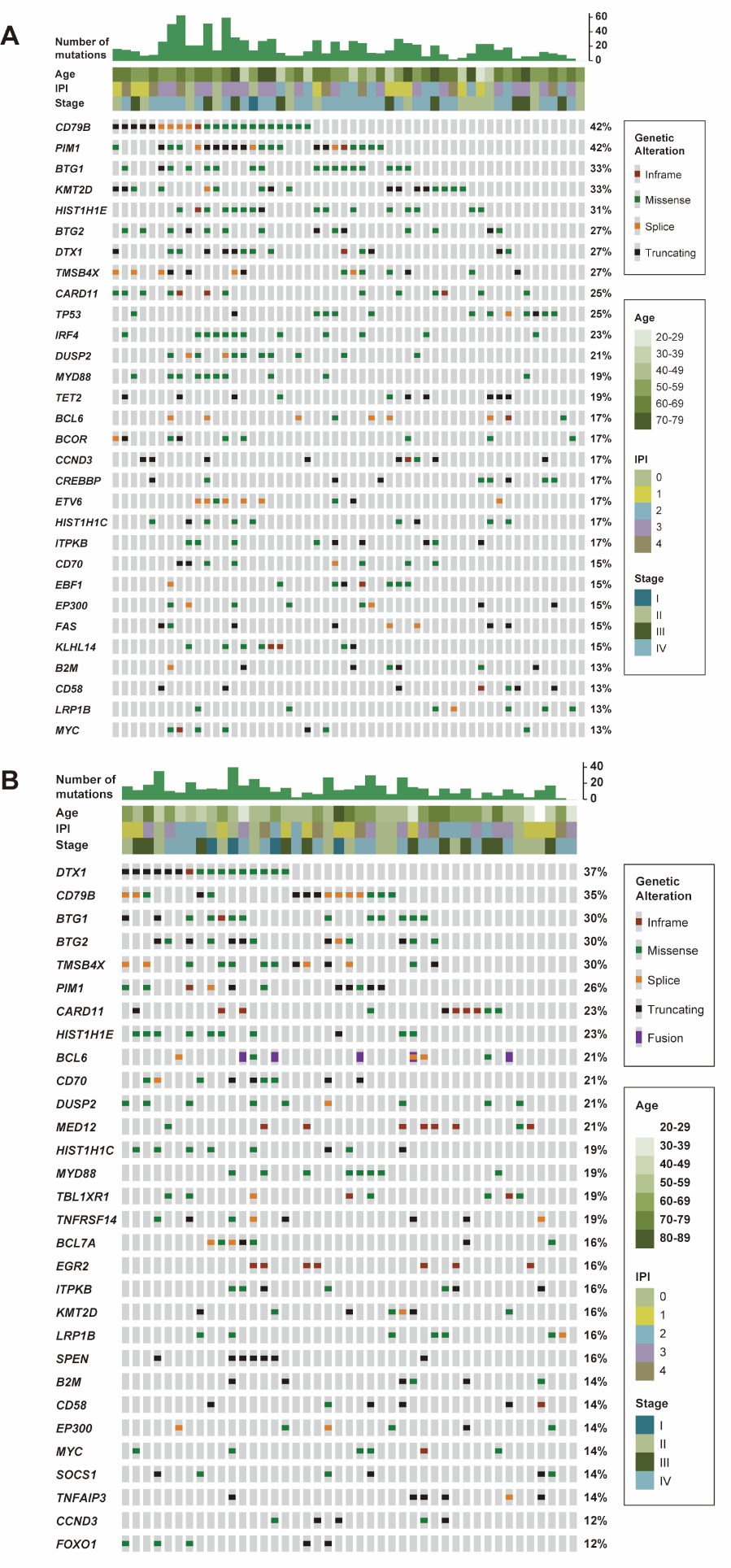


**Fig. S1** Mutation profiles of newly diagnosed DLBCL patients based on (A) plasma ctDNA (N=52) and (B) tumor gDNA (N = 43) in this cohort.


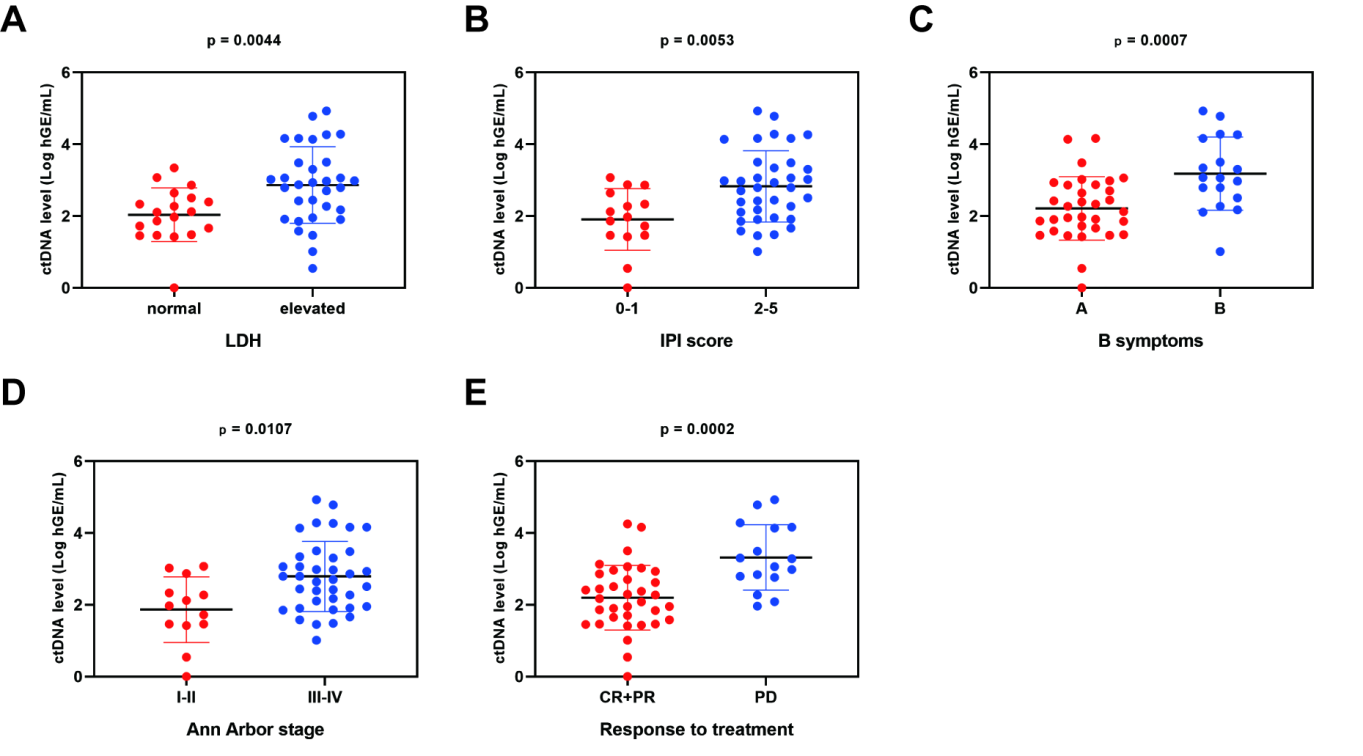


**Fig. S2** Correlation between pretreatment ctDNA levels and 1) known prognostic factors, including baseline LDH (A), IPI score (B), B symptoms (C), Ann Arbor stage (D), 2) the response to the first line treatment (E). Undetectable ctDNA was assigned the value of 0 Log hGE/mL.


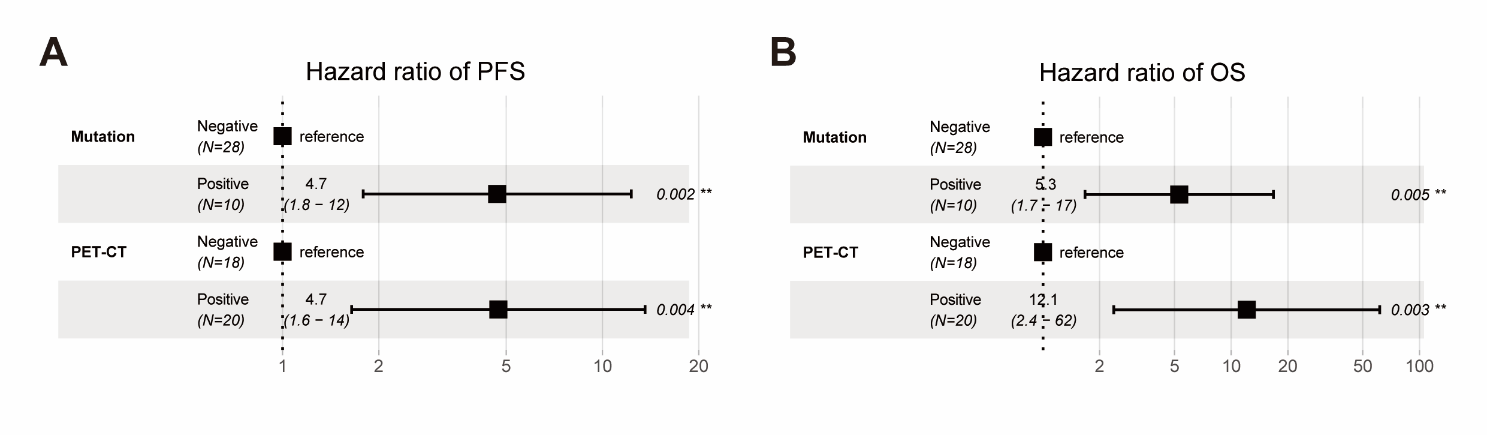


**Fig. S3** Multivariable cox proportional hazard models for PFS and OS based on interim ctDNA and PET-CT.


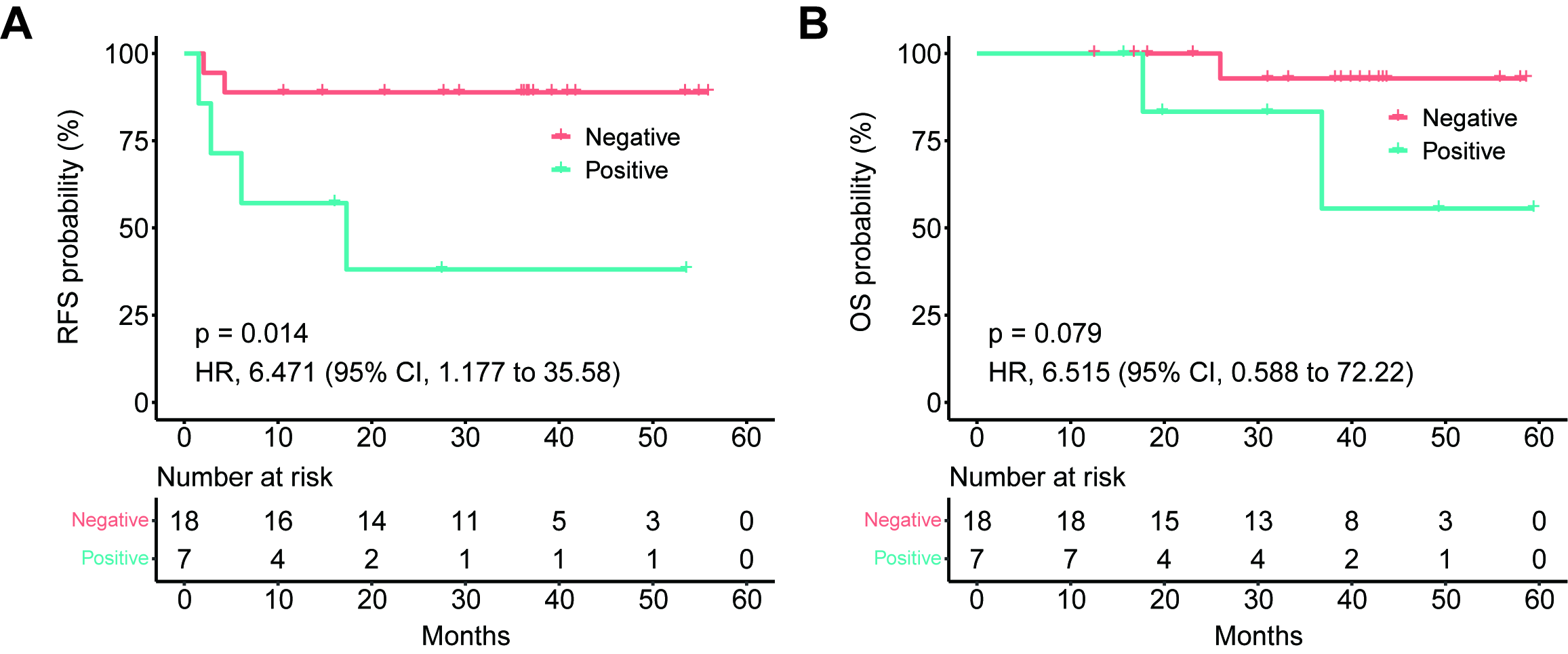


**Fig. S4** Kaplan-Meier estimates of RFS (A) and OS (B) according to the ctDNA-MRD status.
